# Repurposing Clinical Agents for Chemical Exchange Saturation Transfer Magnetic Resonance Imaging: Current Status and Future Perspectives

**DOI:** 10.3390/ph14010011

**Published:** 2020-12-24

**Authors:** Zelong Chen, Zheng Han, Guanshu Liu

**Affiliations:** 1 Medical Imaging Center, Nanfang Hospital, Southern Medical University, Guangzhou 510515, China; chenzlxy@i.smu.edu.cn; 2 Russell H. Morgan Department of Radiology, Johns Hopkins University School of Medicine, Baltimore, MD 21205, USA; zhan14@jhmi.edu; 3 F.M. Kirby Research Center for Functional Brain Imaging, Kennedy Krieger Institute, Baltimore, MD 21205, USA

**Keywords:** CEST, molecular imaging, contrast agents, diamagnetic, MRI, bioorganic

## Abstract

Molecular imaging is becoming an indispensable tool to pursue precision medicine. However, quickly translating newly developed magnetic resonance imaging (MRI) agents into clinical use remains a formidable challenge. Recently, Chemical Exchange Saturation Transfer (CEST) MRI is emerging as an attractive approach with the capability of directly using low concentration, exchangeable protons-containing agents for generating quantitative MRI contrast. The ability to utilize diamagnetic compounds has been extensively exploited to detect many clinical compounds, such as FDA approved drugs, X-ray/CT contrast agents, nutrients, supplements, and biopolymers. The ability to directly off-label use clinical compounds permits CEST MRI to be rapidly translated to clinical settings. In this review, the current status of CEST MRI based on clinically available compounds will be briefly introduced. The advancements and limitations of these studies are reviewed in the context of their pre-clinical or clinical applications. Finally, future directions will be briefly discussed.

## 1. Introduction

In 2000, Balaban and his colleagues demonstrated a new type of MRI contrast could be obtained by a few diamagnetic metabolites containing exchangeable protons and named it as “chemical exchange saturation transfer” (CEST) [1]. To date, CEST MRI has been exploited to detect a broad spectrum of compounds, both endogenously and exogenously. In an endogenous CEST MRI study, no contrast agent injection is required. Rather, it detects the CEST contrast stemming from endogenous molecules, which may change substantially as a result of the changes in the concentrations of biological molecules, intra- or extra- cellular pH, or cell function and metabolism, associated with pathological abnormalities. Indeed, many early CEST MRI studies have been focused on detecting the altered metabolites, protein concentration, and pH in cancer [1,2,3,4,5]. Very often, the exchangeable protons in endogenous molecules, such as proteins, are abundant, hence providing sufficient sensitivity for CEST MRI detection. As such, CEST MRI has become an appealing non-invasive technology to detect and monitor the progression of many diseases, including cancers [5,6,7,8,9], stroke [5,10,11,12,13,14,15], neurodegenerative diseases [16,17,18,19], musculoskeletal diseases [20,21,22,23], and kidney diseases [24,25,26]. Interested readers are referred to several recent reviews covering the development and applications of endogenous CEST MRI [27,28,29,30].

On the other hand, exogenous-agent-based CEST MRI can be designated to target specific molecular targets and biomarkers, thereby potentially providing higher specificity than the endogenous counterparts. By the name, the agent-based approach requires administering contrast agents, which is often referred to as a minimally invasive approach to differentiate from the imaging approaches that are completely non-invasive. Over the last two decades, hundreds of exogenous CEST MRI agents have been reported, which, based on the agent’s magnetic properties, can be categorized into diaCEST, for those use diamagnetic agents [1,29,31], paraCEST, for those use paramagnetic metal complexes [32,33,34], and hyperCEST, for those use compounds containing hyperpolarized atoms [35,36]. Among them, diaCEST agents have the highest biocompatibility and versatility. Mounting evidence shows that diaCEST agents, including both natural compounds and synthetic agents, can be used for a broad spectrum of biomedical applications. More importantly, many clinical compounds can be directly used as diaCEST MRI agents, providing a practical way to pursue highly translatable MR molecular imaging. This review will focus on clinical materials and agents that have potential of being “off-label” used as CEST MRI agents and thereby may have an immediate clinical impact.

## 2. Basics of CEST MRI

The phenomenon of intermolecular saturation transfer through proton exchange was known as early as 1960s [37]. In 1990s, in the context with development of metabolic MR spectroscopy and imaging, chemical exchange saturation transfer NMR and MRI gained a renewed interest because of the ability to detect small concentrations of molecules indirectly by the change in water MR signal [2,3,4,38,39,40], which later was named chemical exchange saturation transfer (CEST) by Ward et al. [1].

In a CEST MRI study, the magnetization of exchangeable protons are first manipulated (i.e., saturation in most of the CEST studies) using radiofrequency (RF) pulses irradiated at the specific frequency offset corresponding to the chemical shift difference between the exchangeable protons and water. For instance, the frequency offsets (∆ω) are around 1.2 and 3.5 ppm (with respect to the water resonance) for hydroxyl protons on glucose and amide protons on peptide and proteins, respectively. As exchangeable protons constantly exchange between the CEST agents and water molecules, the saturated magnetization is transferred continuously from CEST agents to water, resulting in a decrease in water signal (MR image intensity). Although a single exchange-transfer process only produces a water signal decease equivalent to the number of exchangeable protons in the CEST agent pool (i.e., mM here), continuous irradiating at the frequency offsets of the exchangeable protons will pump more and more saturated protons from the CEST pool to bulky water pool (where proton concentration [H]~110 M), resulting in a substantial MR signal change, namely CEST contrast. The CEST technology thus provides a detection amplification strategy allowing detecting a small amount of exchangeable protons through a relatively large change in water MR signal. Especially for protons with relatively fast exchange rate (k_ex_ > hundreds sec^−1^) but within the slow to intermediate regime, this strategy can provide a nearly 1000-time signal amplification [41].

The pulse sequence for CEST labeling is similar to traditional magnetization transfer contrast (MTC) labeling in that a frequency-selective RF saturation pulse (power = B_1_, offset = ∆ω) is applied for a period of time (T_sat_), followed by subsequent MR images acquisition. For a full spectral assessment, a range of offsets are intermittently irradiated, and one image is acquired per offset. Typically, an image without saturation pulses is also acquired as the reference image. The CEST MRI signal is often depicted using Z-spectrum, in which the normalized MR signal (S^Δω^)/S_0_ is plotted with respect to the frequency offset of the saturation pulses (∆ω), where S^Δω^ is the MRI signal with RF irradiated at Δω, and S_0_ is the reference signal acquired without RF saturation. The CEST contrast is commonly quantified using magnetization transfer ratio asymmetry (MTR_asym_), defined by MTR_asym_ = (S^−Δω^ − S^+Δω^)/S_0_, where −Δω is the frequency offsets on the opposite side with respect to the water frequency offset (set to 0). While bearing several limitations, the MTR_asym_ approach can effectively separate the CEST effect from other effects such as water direct saturation and MTC co-existing in the Z-spectrum and still is the most widely used metric in CEST MRI studies. It should be noted that the CEST contrast (MTR_asym_) is strongly affected by acquisition parameters such as field strength (B_0_) [42,43,44], tissue intrinsic T_1_/T_2_ relaxation times [45,46], the shape, B_1_, and length of the saturation RF pulses [45,47,48]. Importantly, it is suggested that B_1_ should be adjusted with respect to the exchange rate of a CEST agent, i.e., optimal B_1_~k_ex_/2π [49]. As a result, different exchangeable protons may have different CEST-B_1_ dependences. Hence, caution has to be taken when correlating the measured CEST contrast with physically meaningful parameters such as agent concentration and exchange rate. Interested readers are referred to several excellent review papers [30,33,43,48,50,51] for more details about the CEST MRI technology.

Compared to conventional MRI contrast agents, CEST MRI agents have a number of unbeatable advantages. CEST MRI has the ability to exploit non-metallic, bioorganic, biocompatible, diamagnetic compounds. As endogenous and exogenous biologically relevant molecules and compounds contain hydroxyl (–OH, 0.8–2 ppm from water), amino (–NH_2_, 1.8–2.4 ppm), or amide (–NH, 3.5–6.3 ppm) groups, they inherently are good candidate CEST agents [27,28,51]. To date, a wide range of diamagnetic compounds (Table 1) have been investigated [52], and many of them, for example, X-ray and CT contrast agents [53,54,55], drugs [56,57,58,59], nutrients and supplements [16,41,60,61,62], and drug carriers [52,63], are clinically available agents. The advantage to use these compounds as CEST MRI agents is unprecedented: they can be used directly in humans, which is one of the most formidable challenges for the clinical use of most newly synthesized contrast agents. Besides the excellent potential of translatability, CEST MRI also has a number of technical advantages. First, unlike metallic agents that can strongly affect the inherent tissue T_1_ and T_2_ properties, CEST agents may be used in conjugation with other MRI methods simultaneously as exchangeable protons only slightly affect tissue T_2_ times and have a negligible effect on tissue T_1_ times. Moreover, CEST MRI contrast can be turned on and off at will by turning RF pulses on and off [64,65]. Hence, it is possible to simultaneously acquire other (inherent) MRI contrast and CEST MRI contrast [66,67], allowing combined detection of CEST agents with other morphologic, functional, and molecular assessments. Finally, simultaneous detection of multiple CEST agents is also possible as long as the agents have distinctive CEST offsets, which sometimes is referred to as multi-colored MRI detection [62,65,68,69].

In the next sections, we will review the recent development of the off-label use of clinical agents and compounds in each category for CEST MR molecular imaging.

## 3. Clinical X-Ray Agents for CEST MRI

**Figure 1:**
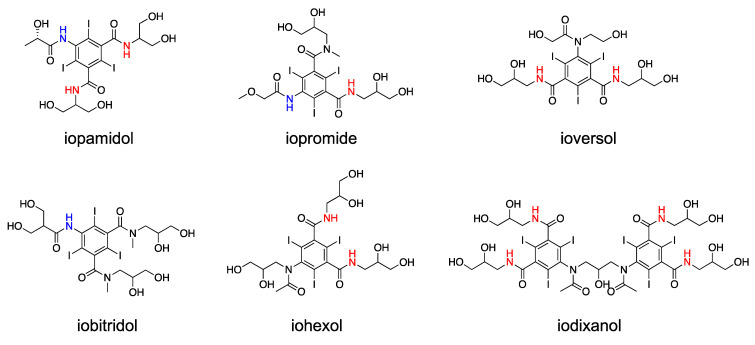
Chemical structures of X-ray/CT contrast agents that have been investigated for CEST MRI studies. Because of the different chemical environments, the amide protons may have different chemical shifts, which are indicated in different colors.

Iodinated X-ray/CT agents are one of the earliest and widely studied clinical compounds for CEST MRI. To date, iopromide (trade name: Ultravist), iopamidol (Isovue), iodixanol (Visipaque), ioversol (Optiray), iobitridol (Xenetix), and iohexol (Omnipaque) have been investigated as CEST agents (Figure 1). These agents are routinely used in the clinic as contrast materials for X-ray/CT scans with well-documented safety profiles. They can be injected to patients intravenously at a relatively high dose, for example, up to 200 mL of iopamidol (~900 mM) [95]. Many iodinated agents contain exchangeable aryl-amide and hydroxyl protons, which have been utilized to shorten the water T_2_ relaxation time, a MRI contrast now named T_2ex_, as early as in 1988 by Aime et al. [96]. Compared to alkyl amide, these aryl amide protons have larger frequency offsets (i.e., Δω~5.2–5.6 ppm) and a much faster exchange rate (*k*_ex_~2560 s^−1^) [97,98], hence providing favorable conditions for detection by CEST.

Clinically, iodinated contrast agents are widely used in dynamic contrast enhanced (DCE) CT scans to improve the visualization and differentiation of normal and abnormal tissues based on their different hemodynamic properties. Similarly, iodinated agents-based CEST MRI were used to detect malignancies [99] and assess perfusion properties in dysfunctional tissues and tumors [100,101,102]. In a recent study, Anemone et al. compared the tumor perfusion parameters measured by both CEST MRI and traditional Gd-based dynamic contrast-enhanced (DCE) MRI by sequentially i.v. injecting iodinated contrast agents (iodixanol, iohexol, and iopamidol) and gadoteridol to mice bearing murine TS/A and 4T1 breast tumors [101]. The results showed a strong correlation between the spatial distribution between iodinated contrast agents and gadoteridol and moderate correlation between the tumor perfusion parameters derived from CEST MRI and those by Gd-DCE MRI. The ability to assess tissue perfusion was also demonstrated in the kidneys, where the altered renal perfusion properties were studied in several animal models of acute kidney injury [100,103] and chronic kidney diseases [102]. Compared to DCE-CT, CEST MRI utilizes the same iodinated agents to characterize tissue perfusion noninvasively, but without any ionizing radiation. However, studies of the direct comparison between the perfusion parameters derived from DCE-CT and those from CEST MRI still lack.

Another advantage of CEST MRI is its ability to measure pH as pH strongly affects the exchange rates of exchangeable protons. The aryl amide protons in iodinated contrast agents also have strong pH dependence, making them suitable for pH measurement. Agents like iopromide [53] and iopamidol [104] contain two different types of amide protons with distinctive frequency offsets (i.e., 4.2 and 5.6 ppm) and different pH-dependencies, which can be utilized to measure pH using a ratiometric approach. In such an approach, the ratio of the two CEST contrasts is calculated to eliminate the effects of agent concentration and tissue T_1_ relaxation times, allowing accurately determining pH as long as sufficient CEST contrasts are present. However, even if only one CEST contrast is present, as shown by several recent studies, the B_1_-dependence of the CEST contrast can also be exploited to estimate pH using the ratiometric of the CEST contrast acquired at different B_1_ strengths. For example, the CEST contrast of iobitridol at 5.6 ppm was demonstrated to measure extracellular pH in adenocarcinoma TSA tumors in mice (4 g I/kg, i.v.) [54].

**Figure 2:**
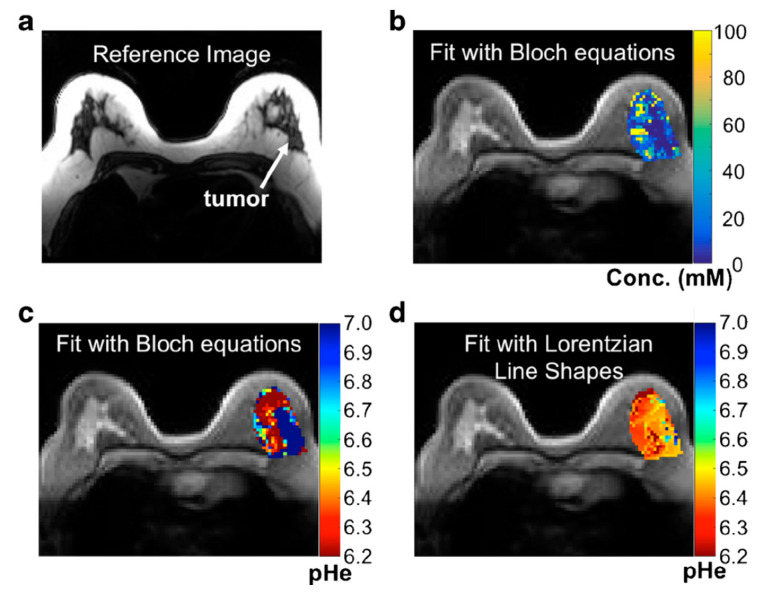
Parametric maps of the patient with high-grade invasive ductal carcinoma. (**a**) A representative image of the patient with high-grade invasive ductal carcinoma. (**b**) A parametric map of tumor concentration determined with Bloch fitting is overlaid on the anatomical image. (**c**) A parametric map of tumor pH_e_ determined with Bloch fitting is overlaid on the anatomical image. (**d**) A parametric map of tumor pH_e_ determined with Lorentzian fitting is overlaid on the anatomical image. (Reprinted with permission from Ref [108]).

To date, mounting studies have explored the utility of iodinated contrast agents in detecting the extracellular pH in different tissues such tumors and kidneys, both preclinically (in mouse models) and clinically. As these agents tend to be confined in either intravascular or extravascular-extracellular space (EES) after administration, the primary use is to map extracellular pH (pH_e_). Altered pH_e_ is considered an important hallmark in many diseases as a consequence of abnormal cellular metabolism, for instance, the Warburg effect in the tumors. Using iopamidol-based CEST MRI, Longo et al. [105] recently showed that the uptake of ^18^F-FDG correlated inversely with pH_e_ in TSA murine breast tumors, indicative of the causal relationship between elevated glucose uptake/glycolysis and acidified pH_e_. It should be noted that the study was carried out using a 3T Bruker preclinical scanner, implying that the pH mapping method might be directly used for clinical studies even though the dose of 4 g I/kg (i.v.) is relatively high. Besides cancer diagnosis and characterization, pH mapping is also a useful tool for monitoring treatment responses noninvasively. For example, using iopromide as the contrast agent, CEST MRI revealed a statistically significant increase in tumor pH_e_ (~0.10 pH unit) within the first day of the treatment of everolimus (RAD001), an mTOR inhibitor, correlating well with the decreased tumor growth rate. When the tumor grow rate resumed at 7 days after the treatment, pH_e_ was found back to be acidic again, strongly indicating pH_e_ measured by CEST MRI can be used as a surrogate biomarker for the therapeutic efficacy of anticancer therapies [106]. In another study, CEST-based pH mapping (using iopamidol) was used to monitor the treatment effect of dichloroacetate, a drug that reverses the glycolysis in the tumor, and showed a good correlation between the alkalization of pH_e_ in early time point (i.e., 3 days post-treatment) and treatment responses [107]. Recently, the performance of iopamidol and iopromide were compared in the context of mapping tumor pH_e_ by Pagel and his colleagues [55]. The study revealed that the two agents had similar performance characteristics and produced pH_e_ values that were not significantly different. Interestingly, iopromide allows pH measurement with a higher dynamic range, while iopamidol produces more precise results. Also, iopromide consistently measured a greater region within the tumor than iopamidol. Currently, several clinical studies or trials (e.g., NCT02380209) are being carried out to investigate the clinical utility of CEST-based pH mapping in a variety of diseases such as metastatic ovarian and breast cancer (Figure 2) [108].

The CEST-based pH mapping has also been applied to other diseases. For example, Pavuluri et al. successfully used iopamidol as the contrast agent to assess the changes in renal pH_e_ associated with methylmalonic acidemia (MMA) [102]. The study revealed a variation in pH, ~0.45 units for severe disease mice compared to 0.06 and 0.01 for moderate disease and healthy controls. High and her colleagues [109] used CEST MRI to assess the pH of joint fluid and tissues in four patients by the means of intra-articular administration of either iopamidol (35 mL, 370 mgI/mL, *n* = 2) or iohexol (35 mL, 350 mgI/mL, *n* = 2). Their study revealed that, on a 3T clinical MRI scanner, the ratio of powers 0.54/1.10 µT showed the strongest correlation with pH. This method holds promise for early detection of the degradation of cartilage and meniscus.

## 4. Nutrients and Supplements for CEST MRI

To date, a wide array of nutrients and supplements have been reported for CEST MRI agents. Those compounds are generally considered as safe and can be directly applied to human subjects. Many of these compounds have been used as non-targeted contrast agents with the generated CEST MRI contrast reflecting the vascular properties and hemodynamic characteristics of the tissues. Some of them are also associated with cellular metabolism, and appear useful for studying the altered metabolism associated with particular diseases.

### 4.1. Glucose and Its Derivatives

**Figure 3:**
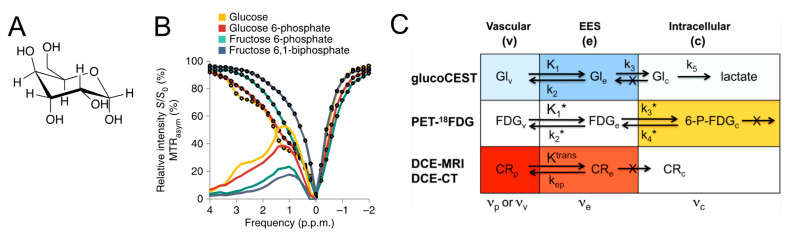
GlucoCEST MRI. (**A**) Chemical structure of glucose. (**B**) Z-spectra and MTR_asym_ spectra of glucose and its first-step metabolite glucose 6-phosphate, as compared to that of fructose 6-phosphate and fructose 6,1-biphosphate (Reprinted with permission from Ref [60]). (**C**) Overview of rate constants and contrast contributions (darker color = higher contrast; white is negligible contrast) for glucoCEST, ^18^FDG-PET, and contrast-enhanced MRI and CT in tumors. For glucoCEST, the glucose concentrations in vascular space and EES are comparable, but due to lower pH the EES has a higher signal contribution. Intracellular signal is very small to negligible due to rapid glycolysis. In PET, the signal is predominantly due to trapped intracellular phosphorylated FDG. For contrast-enhanced MR and CT, the agents occupy only plasma in blood and while they enter the interstitium, the EES concentration is generally lower than in plasma due to limited K^trans^. Glucose, on the other hand moves freely into the interstitium and the erythrocytes. v = vascular (plasma + erythrocytes), p = plasma. Reprinted with permission from Ref [61].

Glucose, also called dextrose, probably is the most widely studied CEST MRI agent. Glucose is a monosaccharide with the molecular formula C_6_H_12_O_6_, and its pyranose form (Figure 3A, the dominant form of glucose molecule in aqueous solution) containing five fast exchangeable hydroxyl (–OH) protons. Glucose is an essential nutrient and serves as the primary metabolic fuel for almost all organs, including brain, placenta, and fetus. d-glucose is available in intravenous injection solution in the clinic for treating hypoglycemia by providing carbohydrate calories to a person who cannot eat because of illness, trauma, or other medical conditions, and testing for glucose tolerance. Since 2012, d-glucose has been explored as a safe CEST MRI contrast agent for detecting breast tumors [61] and brain tumors [60] in murine models. Studies showed that d-glucose can generate a broad, strong CEST contrast between 0.8–2.2 ppm (peak position ~1.2–1.3 ppm) and a weak signal at around 2.8 ppm (Figure 3B). Of note, the exact peak position of glucose highly depends on the B_1_ strengths used. Due to the fast exchanging nature of hydroxyl protons, the CEST contrast of glucose is highly sensitive and inversely related to pH, decreasing with increasing pH in the range between pH 6–8. In both the very first studies [60,61], the glucose-enhanced CEST (glucoCEST) contrast in the tumor was found different from that of ^18^FDG-PET, attributable to the difference in cellular metabolism between FDG and d-glucose (Figure 3C). Glucose can be converted to lactic acid and other metabolites by glycolysis in fast-growing tumor cells, resulting in a quick elapse of glucoCEST signal right after glucose enters the cells. Indeed, it has been debated which compartments are the major contributors of glucoCEST signal for a long time. Three compartments are involved after glucose is intravenously injected: intravascular space, extravascular extracellular space (EES), and intracellular space. The transport of glucose is rather rapid; it can quickly extravasate via glucose transporters (e.g., GLUT-1), followed by quick uptake by cells where it is quickly metabolized. As a result, the exact contribution of each compartment to the overall CEST contrast has not been exactly measured, while EES, which is often relatively large (30–40%) and acidic in the tumor, is likely the dominant contributor. For example, studies have shown that, in the brain tumors, the increase of glucoCEST signal correlates well with the changes in cerebral blood volume, glucose transporter, and BBB integrity [70,110,111]. GlucoCEST contrast has been suggested as an imaging biomarker for tumor aggressiveness [61] and inflammatory responses in the kidney [112] and placenta [113].

Currently, most glucoCEST MRI studies were performed using a dynamic imaging scheme, in a similar way to DCE MRI, namely dynamic glucose enhanced (DGE) CEST MRI. This is at least partially because the offset of glucose falls in the range that enormous endogenous CEST MRI background exists. The dynamic acquisition scheme can effectively improve the contrast-to-noise ratio and thereby the specificity of glucoCEST detection. DGE CEST MRI can be acquired using either a single offset approach [70,114] or on-resonance approach [115,116], with both having the ability to provide a temporal resolution of seconds.

**Figure 4:**
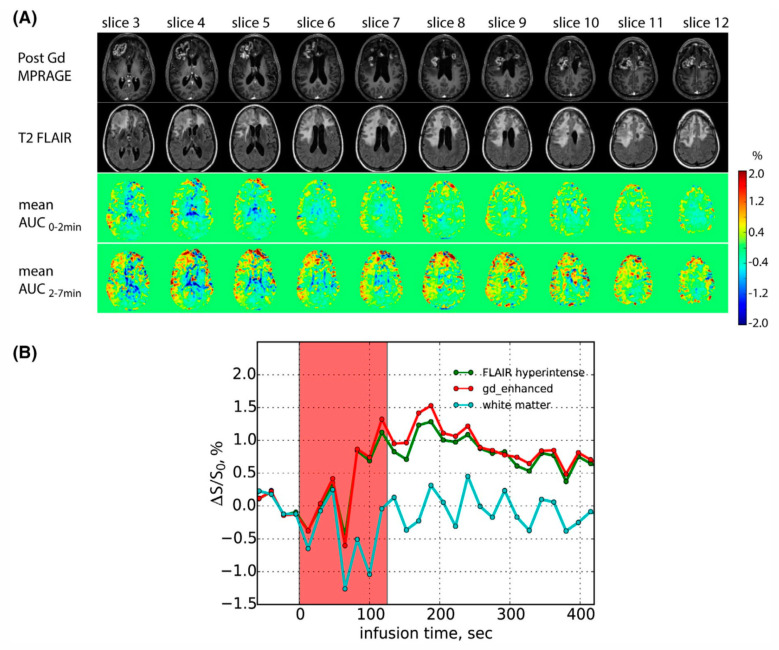
Glucose-enhanced CEST MRI (GlucoCEST) study using a 3 Tesla human scanner. (**A**) Post-Gd T_1_ MPRAGE, T_2_ FLAIR, mean DGE AUC (0–2min), mean DGE AUC (2–7 min) images; and (**B**) DGE signal as a function of infusion time in the Gd-enhanced, FLAIR hyperintense, and posterior normal appearing WM. The AUC maps clearly show the higher uptake of glucose in the regions with hyperintense in the T2FLAIR images than brain parenchyma. The patient was previously diagnosed with an IDH mutant glioblastoma at the time point of 3 months post-surgery. Reprinted with permission from Ref [117].

GlucoCEST MRI has been translated from preclinical studies to clinical studies in recent years. For example, the first human study was reported in 2015 by Xu and her colleagues on a 7T clinical scanner [114]. The feasibility to perform glucoCEST MRI in humans using 3T clinical scanners has been confirmed by different research groups (Figure 4) [115,117]. Given glucose is considered a very safe agent even for patients with impaired kidney function or pregnant women, a broad clinical application of glucoCEST MRI is anticipated.

**Figure 5:**
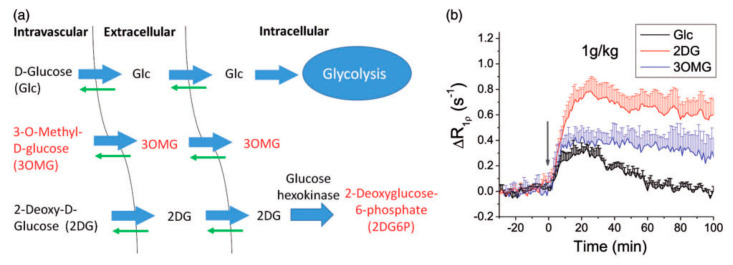
In vivo characteristics of intravenously injected 3OMG, Glc and 2DG. (**a**) Three-compartment schematic illustration of transport and metabolism of Glc, 2DG, and 3OMG in normal brain. Intravascular, extravascular–extracellular, and intracellular pools are considered. Molecules with red label indicate non-metabolizable. (**b**) Glucose or glucose analogs were intravenously injected into normal rats. Rat brain R1*ρ* changes by 1 g/kg administration of d-glucose (Glc, n = 4), 2DG (*n* = 4) and 3OMG (*n* = 5) were plotted, where the injection time is indicated by the gray arrow. (Reprinted with permission from Ref [121]).

From the MRI contrast agent perspective, while being very safe, glucose has the drawback to be rapidly metabolized inside most cells, making the contrast disappearing completely in several minutes. To overcome this inherent drawback, a number of glucose derivatives, including 2-deoxy-d-glucose (2-DG) [74,75,76], 3-*O*-methyl-d-glucose (3OMG) [71,72], glucosamine [81] and dextrans [77,78,118,119], have been reported. Those compounds have similar CEST contrast as glucose, but different transport and metabolic properties (Figure 5). Regarding this aspect, a recently published review paper is suggested for further reading [120].

### 4.2. Pharmaceutical Excipients

A number of natural products and substances can be used in formulating medicines, namely pharmaceutical excipients. They are biological inactive but can contribute to product attributes such as stability, biopharmaceutical profile, appearance and patient acceptability, and the ease of product manufactory. Excipients are pharmacologically and toxicologically inactive, allowing them to be used at high doses. Many these excipients contain hydroxyl, amine, amide, and/or other types of exchangeable protons, making them inherently CEST MRI detectable. Hence, drug excipients can be used to indirectly monitor the delivery of the active drugs that those excipients are used along. The first investigation of the CEST MRI detection of pharmaceutical excipients was conducted by Longo et al. [80], where five commonly used excipients (sucrose, *N*-acetyl-d-glucosamine, ascorbic acid, meglumine and 2-pyrrolidone) were characterized in vitro and later used in mouse tumor models. Among the compounds studied, meglumine exhibited the strongest contrast enhancement (ΔCEST > 5%) in both B16 and TS/A models by its CEST contrast at 3.5 ppm, attributed to amide protons. As excipients have extremely good safety profile and have been extensively tested in humans, they are excellent candidates of CEST contrast agents ready for clinical translation.

### 4.3. Biopolymers

Several clinically available polymers have also been reported as CEST MRI contrast agents. For example, our group has been focusing on exploiting dextrans as a platform agent that is highly translatable and sensitive for pursuing MR molecular imaging. Dextrans are glucose polymers produced by bacteria from sucrose or by chemical synthesis, and therefore only available as exogenous agents. Dextrans consist of glucose units polymerized predominantly through α-1,6-glucosidic linkage (~95%) and 1,3-linkage (~5%) [122]. Dextrans are available in multiple molecular weights ranging from 1 kDa to 2000 kDa (particle diameters from 1 to 54 nm, respectively) [123,124]. As a group of important clinical materials, the pharmacokinetics of dextrans of different molecular weights have been well studied. Large dextrans (>40 kDa) are excreted poorly from the kidney, remaining in the body for weeks, while small dextrans (<20 kDa) are quickly cleared from the body [125]. Dextrans have been used clinically for more than 6 decades as plasma volume expanders, peripheral flow promotors, and anti-thrombolytic agents with a proven safety profile [126,127]. Dextran 70 (dextran with MW~70 kDa) is on the WHO Model List of Essential Medicines. Non-labeled dextrans of different MW can be directly used as MRI agents to probe tissue permeability in different size ranges [77] as well as the changes in tumor vascular permeability in response to anti-vascular treatment [118]. Dextrans can also be easily conjugated with targeting ligands for targeted imaging of tumor specific biomarkers, for example, prostate-specific membrane antigen (PSMA) in prostate tumor cells [78] and extradomain-B fibronectin (EDB-FN), a tumor microenvironmental biomarker overexpressed in pancreatic tumors [119].

Another example is poly-l-glutamate as demonstrated by Harris et al. [63]. Poly-l-glutamate is a nontoxic polymer that is being investigated as a drug carrier for treating cancer. While poly-l-glutamate is not CEST MRI detectable, its metabolic product glutamate is CEST MRI detectable. In tumor cells, poly-l-glutamate can be cleaved by the lysosomal enzymes, such as cathepsins, into glutamate moieties, and subsequentially be detected by CEST MRI at 3 ppm.

### 4.4. Biologically Active Molecules and Metabolites

There is a large cohort of biological molecules and metabolites that have CEST MRI signals. For example, Ryoo et al. developed CEST-based analytical methods to accurately quantify hydrogen peroxide (H_2_O_2_), an important biological molecule involved in redox processes, cell signaling pathways, oxidative stress, and inflammation [128,129]. The study showed that H_2_O_2_ has a unique CEST offset at ~6.2 ppm and could be detected by CEST MRI with more than 1000 times signal amplification compared to a conventional NMR approach [41]. Metabolites have also been studied extensively, presumably as endogenous indicators for neurodegenerative diseases and cancer. For example, Chan et al. measured 15 common cellular metabolites in a panel of differentially aggressive human breast cancer cell lines and showed that creatine, myoinositol, glutamate, and glycerophosphocholine contribute significantly to the apparent CEST contrast of the tumor cells with all of them negatively correlated with breast cancer aggressiveness [130]. Potentially, many of those agents can be used as exogenous CEST contrast agents. In a recent study, Shin et al. successfully developed urea as an exogenous CEST contrast agent for quantitative imaging of the spatially varying urea concentrating capacity of the kidney and, hence, monitoring renal function [131].

## 5. Clinical Drugs for CEST MRI

Probably the most desirable way to construct theranostic systems is to make the drugs to be delivered imageable, e.g., by labeling the drugs with radioisotopes. Only a handful of drugs can be directly imageable, for example, if containing fluorophores (such as doxorubicin [132]) or ^19^F atoms (such as 5-fluorouracil, 5-FU [133]). Most commonly used drugs cannot be imaged using conventional MRI methods (except MR spectroscopic imaging) until CEST MRI was invented. Given many drugs indeed contain one or more types of exchangeable protons, they in principle can be directly detected by CEST MRI. To date, we and other groups have screened a wide range of drugs and demonstrated their utility in image-guided drug delivery and theranostics in a label-free manner (Table 2). While most of these studies focused on anticancer drugs, a few drugs for neurodegenerative diseases, cardiovascular diseases, and inflammation were also reported.

### 5.1. Anticancer Drugs

**Figure 6:**
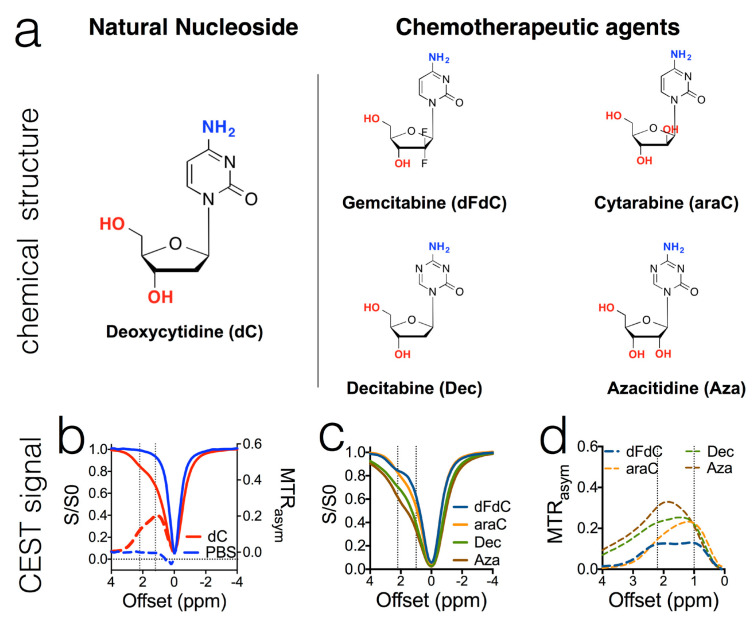
The chemical structure of cytidine-based agents (**a**) and their CEST MRI contrast, as shown both by z-spectra (**b**,**c**) and MTR_asym_ plots (**b**,**d**). All samples were prepared in PBS (pH 7.4) at a concentration of 20 mM and measured at 37 °C using a 3.6 μT, 3 s CW RF pulse. Reprinted with permission from Ref [59].

In one of our early studies, we selected a library of 22 anticancer drugs and characterized their CEST properties [59]. The results showed that pyrimidine analogs (Figure 6), purine analogs, and antifolates can be used as CEST contrast agents if the concentration is sufficiently high. For example, gemcitabine, a first-line chemotherapeutic drug for several types of solid tumors including pancreatic cancer, exhibits strong CEST contrasts at both 2.2–2.3 ppm and 1.0 ppm, attributable to amine (NH_2_) and hydroxyl exchangeable protons, respectively. Similar to gemcitabine, most pyrimidine analogs are also able to generate CEST contrast, however with slightly different offsets and quite different sensitivity. For example, as shown in Figure 6C,D, when pyrimidine is replaced by triazine, the NH_2_ protons of decitabine (Dec) and azacitidine (Aza) showed 2–3 times stronger CEST effects (i.e., MTR_asym_ (2.3 ppm) = 0.23 and 0.31 per 20 mM agent, respectively) than that of deoxycytidine (i.e., MTR_asym_ (2.1 ppm) = 0.12 per 20 mM agent) [59]. Since then, the list of CEST imageable drugs has been expanding, and anti-cancer drugs in different categories were also reported, such as DNA alkylating agent (melphalan) [57], DNA methylation inhibitor (olsalazine) [134], and photosensitizer (porphyrins and chlorin) [135]. Among them, some agents have very desirable CEST properties, i.e., offsets far from the majority of endogenous metabolites (0–4 ppm), which may allow more specific detection and longitudinal assessment of not only drug delivery and but also drug action (e.g., drug metabolism and the interaction of a drug with its targeted molecules and cells). For example, olsalazine has a large downfield CEST contrast at ~9.8 ppm from the water resonance [134], and porphyrins and chlorins have unusual CEST peaks at −8 to −13.5 ppm [135]. As a result, in vivo CEST MRI detection is more specific and allows “multicolor” MRI detection of multiple agents [62,91].

As a relatively high concentration is required to generate sufficient CEST contrast, to date, most of these studies were performed using their nanoparticulate forms. Drugs can be either encapsulated in nanoparticles or used as building blocks of nanoparticulate drug delivery systems. Using the later strategy, nanofiber hydrogel [56] and enzyme-activable self-assembling nanoparticles [134] were constructed to accomplish CEST theranostics, by which one can easily and unbiasedly assess drug delivery in a label-free manner without extra chemical labeling.

Several studies also showed that, besides drug delivery and distribution, namely pharmacokinetics, the intrinsic MRI signal of a drug can also be used to assess the drug action (pharmacodynamics). For instance, when the prodrug 5-fluorocytosine is converted to 5-fluorouracil by the activity of cytosine deaminase (CDase), the CEST contrast at ~2 ppm (aniline protons) disappears [87], allowing noninvasive assessment of the conversion of prodrug to the effective drug using CEST MRI. Recently, we also applied this strategy to the noninvasive assessment of the activity of deoxycytidine kinase (DCK) [141], a key enzyme responsible for the activation of a broad spectrum of nucleoside-based chemotherapy drugs (e.g., gemcitabine) and low DCK activity is one of the most important causes of cancer drug-resistance. By the activity of DCK, drugs will be “trapped” inside tumor cells, leading to a higher concentration and longer retention time of these drugs in the tumor, and thus an observable increase in CEST contrast in the delayed phase. Otherwise, the CEST contrast will disappear because of no drug accumulation. Based on this principle, we developed a CEST MRI method to detect DCK activity using its natural substrate deoxycytidine (dC) as the imaging probe [141]. Simply by assessing the dynamic CEST contrast changes in the tumor followed by the injection of dC (2 g/kg or 8.8 mmol/kg b.w.), tumor DCK activity can be detected, allowing assessing tumor resistance and predicting treatment efficacy.

### 5.2. Anti-Inflammation Drugs

**Figure 7:**
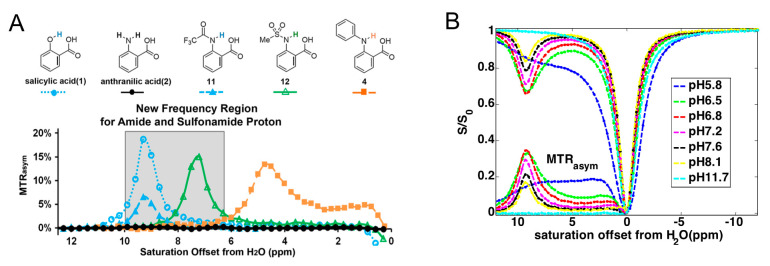
CEST MRI contrast of salicylic acid (**A**). CEST contrast curves for representative salicylic acid (1) and anthranilic acid derivatives (2, 4, 11 and 12) at concentrations of 25 mM (pH 7.1–7.4) using B_1_ = 3.6 μT, T_sat_ = 3 s. The gray box indicates this group of agents includes a new frequency region for amide and sulfonamide protons. Reprinted with permission from [136]. (**B**) pH effect on the contrast of salicylic acid at pH = 5.8–11.7. Concentration = 25 mM, and B_1_ = 7.2 μT. Maximal contrast was observed between pH 6.5 and 7.0. Reprinted with permission from Ref [91].

Salicylic acid (SA) is an important active metabolite of aspirin (acetylsalicylic acid, ASA), a commonly used nonsteroidal anti-inflammatory drug (NSAID). Aspirin is rapidly hydrolyzed (serum t_1/2_ ~20–30 min) in plasma to SA. SA itself is also used widely as a key ingredient in topical anti-acne products. Yang et al. first reported that the phenol proton of SA exhibited an unusually large CEST offset at 9.3 ppm [91]. As shown in Figure 7A, this large chemical shift is attributed to the strong intramolecular hydrogen bonding between phenol protons and their nearby (deprotonated) carboxylic anion. As a result, the CEST contrast of SA is sensitive to pH, decreasing substantially at either low pH (<6) where carboxylate becomes protonated, or at high pH (>11), where phenols are deprotonated. Thanks to the favorable large chemical shift, SA has been extensively studied as a CEST agent alone or as a building blocks for constructing nanoparticles [142,143] and enzyme-responsive agents [144,145,146,147]. In a recent study, Song et al. also demonstrated the utility of SA as an MRI contrast agent for depicting the accessible regions to agents that are intra-arterially injected to the brain and detecting the resulting BBB opening [148]. Interestingly, this study also showed that, when injected through a catheter inserted into the internal carotid artery (ICA), hyperosmolar SA solution can be used to induce BBB opening, making SA a dual functional theranostic agent.

### 5.3. Drugs for Neurodegenerative Diseases and Cardiovascular Diseases

**Figure 8:**
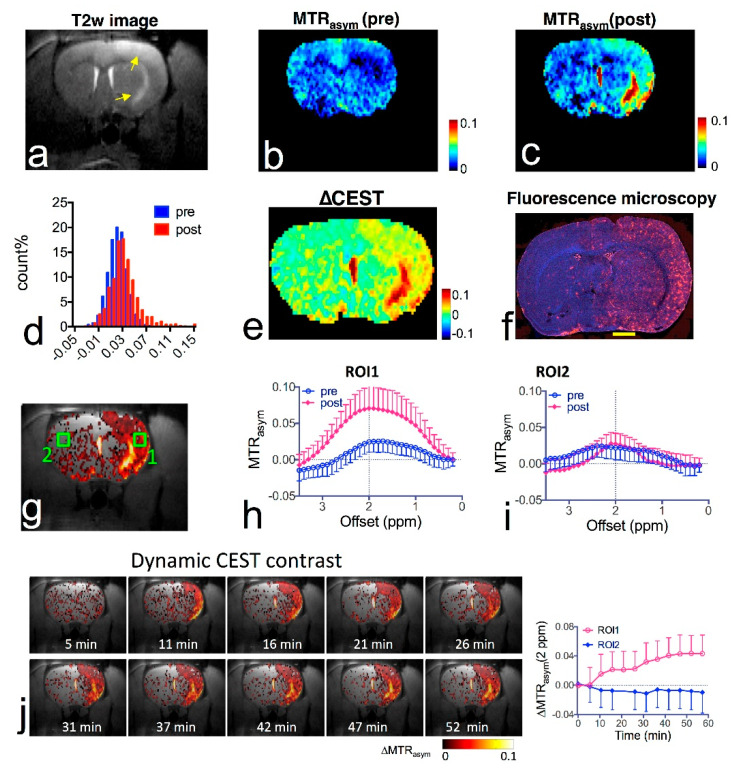
In vivo CEST MRI detection of the delivery of citicoline- liposomes in rat brain after acute ischemic injury. (**a**) T_2_w MRI showing hyperintensity in the injured areas. (**b**) CEST images (MTR_asym_ maps at 2.0 ppm) before and (**c**) at 1.5 h after i.a. injection of CDPC-lipo, showing the elevated CEST contrast in the ischemic region. (**d**) Histogram of the CEST contrast before and after administration. (**e**) ∆CEST map as calculated by ∆MTR_asym_ = MTR_asym_(post) − MTR_asym_(pre). (**f**) Fluorescent microscopy of nuclei (blue, stained with DAPI) and liposomes (red, rhodamine-B-labeled). Scale bar = 2 mm. (**g**) Overlay image of the ∆CEST map and the T_2_w image with the two ROIs chosen for ROI analysis. (**h**) MTR_asym_ plots before (blue) and (**i**) at 1.5 h after (red) i.a. injection of CDPC-lipo for ROI 1 and ROI 2, respectively. (**j**) The dynamic CEST contrast change of the stroke within the first 1.5 h after i.a. injection of CDPC-lipo. Left: dynamic CEST images calculated by The dynamic CEST contrast was quantified by ΔMTR_asym_(t) = MTR_asym_(t) − MTR_asym_(t = 0); Right: the mean ROI CEST contrast change of the two ROIs shown in g, where data are presented as mean ± standard deviation. Reprinted with permission from Ref [58].

Citicoline (CDPC) is a natural supplement with well-documented neuroprotective effects. It has been extensively tested in both preclinical and clinical studies for glaucoma, stroke, and neurodegenerative diseases, such as mild vascular cognitive impairment, Parkinson’s disease, and Alzheimer’s disease [137]. We have shown that citicoline has two inherent CEST contrasts at 1 and 2 ppm, attributed to hydroxyl and amine protons, respectively [58]. Utilizing the inherent CEST contrast of citicoline, both the delivery and distribution of liposomal citicoline in the stroke area were detected by CEST MRI in a rat brain model of unilateral transient ischemia induced by a two-hour middle cerebral artery occlusion [58]. As shown in Figure 8, when administered intra-arterially, non-targeted liposomal citicoline preferentially accumulated in the ischemia affected area, presumably undergoing BBB disruption, which was confirmed by immunofluorescent staining. This study also showed that, liposomes conjugated with anti-vascular cell adhesion molecule (VCAM)-1 antibody effectively improved the inflammation-targeted delivery of citicoline in a delayed time window. Compared to non-targeted IgG-conjugated liposomes, VCAM-1-targeted liposomes can significantly increase concentration of CDPC even injected at 24 h after the onset of stroke. The results demonstrated the great potential to develop label-free CEST theranostic systems for detecting and treating ischemic stroke by utilizing the CEST contrast of the neuroprotective agent citicoline [58].

Another example is acebutolol, a β-adrenergic receptor-blocker commonly used to treat hypertension and arrhythmia. In the liver and intestines, acebutolol is first metabolized to acetolol by carboxylesterases and subsequently to diacetolol by N-acetyltransferases. Patients with N-acetyltransferases deficiency would develop serious adverse effect such as lupus erythematosus (DILE) [138]. Acebutolol is an N-aryl amide derivative and contains exchangeable amide proton at 5 ppm [149]. Therefore, CEST MRI can be used as a practical way to detect the conversion of acebutolol to acetolol and predict acetolol-related toxicity. Our recent study demonstrated that the inherent CEST contrast of acebutolol at 5 ppm can be used to detect esterase-catalyzed hydrolysis of acebutolol in vitro [149].

## 6. Technical Hurdles and Possible Development that Will Ease the CEST MRI Detection and Translation

Despite the great potential of many clinical materials and agents as the next generation MRI contrast agents, it should be noted that several technical hurdles exist, and further technical development is required to clear these obstacles in order to advance the CEST MRI of these clinical materials and agents into real clinical uses.

First, CEST MRI is an inherently insensitive method with a typical detectability on the order of hundreds of µM to mM for most agents. This would impose difficulties for many biologically active drugs because the concentration to be a CEST MRI contrast agent is higher than the pharmacological effective concentration of the drug (~nM–µM). Therefore, CEST MRI detection may be limited to drugs or biologically inactive agents that can be used at a relatively high dose in patients. Fortunately, some drugs can be used at a very high dose. For example, gemcitabine has a clinically suggested dose of 1000 mg/m^2^ body surface area for treating of a variety of cancer types (equivalent mouse dose = 333 mg/kg). In fact, it was reported that a single dose of 800 mg/kg (i.p.) could result in an accumulation of several mM gemcitabine in experimental murine hepatomas [150]. Cytarabine (araC), another commonly used chemotherapeutic drug, can be administered using an even higher regime for treating leukemia, i.e., 3 g/m^2^ (over three hours) for up to eight doses (total dose, 24 to 30 g/m^2^) [151]. As a reference, a single dose of 3 g/m^2^ corresponds to 80 mg/kg and 1000 mg/kg in humans and in mice, respectively. On the other hand, many aforementioned compounds are biologically inactive and they are relatively easier to translate. Biopolymers such as dextrans can provide much higher CEST MRI detectability as the number of exchangeable protons per molecule is substantially high compared to small molecular agents. For example, a molecule of 70 kDa dextran contains approximately 400 glucose units, corresponding to about 1200 hydroxyl protons [77].

Even for drugs that cannot be administered at a high dose due to toxicity issues, CEST MRI may still be applicable to the detection of their nanoparticulate forms. As we and others have demonstrated [62,152,153,154], nanoparticles encapsulated with drugs can provide sufficient CEST contrasts as a result of the high local drug concentration. For example, liposome encapsulation can markedly improve the detection limit from mM (per molecule) to nM (per particle) [62]. Moreover, by modulating the exchange rate between the intra- and extra- liposomal water molecules, liposome encapsulation may be used as an effective way to enhance the CEST contrast of the encapsulated diaCEST agents [155]. Nevertheless, using CEST MRI to detect drug in their nanoparticulate forms is well in line with the development of nanotherapeutics.

Secondly, to date, most of the exogenous agent-based CEST MRI studies were performed using high-field small-animal MRI scanners (e.g., 4.7 T, 9.4 T, or 11.7 T). The translatability of these agents to low field strength clinical MRI scanners still needs further investigation. Several technical challenges exist, such as low SNR, shorter T_1_, and narrower frequency separation from water resonance. Hence, technical development and prospective studies of the translation of CEST agents from high field preclinical scanners to 3T clinical scanners is warranted. Recently, all major MRI manufacturers have developed CEST MRI pulse sequences for detecting endogenous CEST contrast in humans [156,157], and Philips has made its CEST MRI product on market. To date, CEST acquisitions in humans have been implemented with multiple slices [156,158] or with 3D [159], and many advanced fast imaging techniques, such as compressed sensing and parallel imaging [160,161,162], have been used to improve the speed and SNR of CEST detection. The reduced SNR and CNR at low fields could be compensated either by using advanced CEST methods or by increasing voxel size. Also, as evidenced by recent human CEST studies where shaped saturation pulses were used [163,164,165,166], SAR is much reduced at 3 T than high field scanners because SAR is proportional to the square of the field strength [167,168]. Within the last five years, two exogenous agents, D-glucose and iopromide, have entered human testing on 3 T clinical scanners. We expect that many of the abovementioned diamagnetic CEST agents may be translated to the clinic very rapidly.

Finally, the apparent CEST contrast is strongly affected by multiple factors, such as B_1_, T_sat_, concentration, temperature, and pH. Therefore, rigorous quality control and standardized quantification is urgently needed in order to advance CEST MRI to the clinic. Regarding this aspect, both acquisition parameters and analysis methods should be optimized and standardized at least for some prototype protons, e.g., amide protons of proteins, hydroxyl protons of glucose and its derivatives, and amine protons of glutamate, and guanidinium protons of creatine and phosphocreatine. The repeatability on different MRI scanners and test-retest reproducibility on the same MRI scanners should be carefully investigated and reported. It is also helpful to carry out multi-center evaluations using the same set of phantoms (preferably prepared independently) or patients across different MRI vendors. Once these studies are completed, a position paper detailing the standardized (or at least suggested) acquisition and analysis procedures should be published as the guideline for future preclinical studies and clinical translation.

## 7. Conclusions

CEST MRI is a rapidly developing technology with the unprecedented ability to directly use a broad spectrum of clinical agents and even drugs as MRI contrast agents, providing a practical way to realize “label-free” theranostics. The inventory of CEST agents keeps expanding. It is anticipated that many CEST agents may be advanced to the clinic in the near future to help diagnosis or treatment monitoring in a personalized manner.

## Figures and Tables

**Table 1:** DIACEST library (Reprinted with permission from Ref [52]).

Exchangeable Proton	Signal Frequency Offset Δω (ppm)	Examples
Hydroxyl (–OH)	0.8–2, 4.8	Glucose [60,61,70]; 3-OMG [71,72,73] 2DG [74,75,76]; dextran [77,78]; sucralose [79]; sucrose [80]; glucosamine [81]; phenols [82]
Amide (–NH)	3.5, 4.2, 5.6	Poly-L-lysine [83]; iopamidol [84]; iopromide [55]; mobile proteins [5]
Amino (–NH_2_)	1.8–2.4	L-arginine [62,85]; protamine [86]; cytosine/5-FC [87]; proteins [88] folate acids [59]
Heterocyclic ring amide (–NH)	5–6.3	Barbituric acid [86]; thymidine [89]; uridin70e [90]
Hydrogen bonds	6–12	Salicylic acids [91]; imidazoles [92]; H_2_O_2_ [41]
Aliphatic protons (rNOE)	−1.6, −3.5	Mobile proteins [93,94]

Abbreviations: 3-OMG: 3-O-methyl glucose; 2DG: 2-deoxy-d-glucose; rNOE: relayed nuclear Overhauser effect.

**Table 2:** Examples of drugs that can be off-label used drugs as CEST agents.

Category	Examples	Exchangeable Protons
Anticancer drugs	Gemcitabine [59], Cytarabine [59], Decitabine [59], Azacitidine [59],	OH, NH_2_
	Fludarabine [59]	OH, NH_2_
	Methotrexate [59], Pemetrexed [56]	NH_2_, Heterocyclic ring amide
	Melphalan [57]	NH_2_
	Olsalazine [134]	OH
	Porphyrins (TPPS4) [135]	inner nitrogen protons (NH)
Nonsteroidal anti-inflammatory drugs	Anthranilic acid (Flufenamic acid) [136]	NH (hydrogen bond)
	Salicylic Acid [91]	OH (hydrogen bond)
Neuroprotective drugs	Citicoline [137]	OH, NH_2_
Cardiovascular drugs	Acebutolol [138]	*N*-aryl amide
Therapeutic bacteria and virus	Clostridium-NT [139] Oncolytic herpes simplex virus (HSV) [140]	Bacterial cells lysine-rich protein (LRP) gene
